# A Case Report of an Incidental Finding of a Duplicated Inferior Vena Cava in a Patient With Persistent Lower-Extremity Swelling

**DOI:** 10.7759/cureus.113789

**Published:** 2026-08-01

**Authors:** Luis A Santiago-Sulsona, Carlos Diaz-Sepulveda, Sebastian Castaner-Colberg, Rafael Santini, Jorge Martínez-Trabal

**Affiliations:** 1 General Surgery, Centro Médico Episcopal San Lucas, Ponce, PRI; 2 Surgery, Centro Médico Episcopal San Lucas, Ponce, PRI; 3 General Surgery, St. Luke's Episcopal Medical Center, Ponce, PRI; 4 Vascular Surgery, St. Luke's Episcopal Medical Center, Ponce, PRI

**Keywords:** congenital vascular malformation, deep venous stenting, deep venous thrombosis (dvt), ivc anomalies, venogram

## Abstract

Duplication of the inferior vena cava (IVC) is a rare anomaly that arises from abnormal venous development during embryogenesis, leading to two venae cavae instead of one. Although it is usually a clinically silent anatomical variant, this developmental anomaly may alter venous flow within the major venous vessels and cause pathological consequences. Despite being a rare cause of lower-extremity venous disease, it should be included in the differential diagnosis when evaluating a patient with venous disease of no obvious etiology. In this report, we describe an incidental finding of a duplicated IVC in a patient with a 14-year history of persistent left lower-extremity swelling.

## Introduction

The inferior vena cava (IVC) is the primary venous structure responsible for drainage of the lower body. It carries venous blood from the lower extremities, kidneys, pelvis, and abdominal viscera to the heart [1]. Duplication of this important structure is a rare anomaly, with a reported prevalence of up to 3%. Most reported cases of IVC duplication have been incidental findings [2], but this anomaly may be clinically significant in patients with persistent venous disease and no clear etiology [3]. It should therefore be considered when evaluating a patient with deep vein thrombosis (DVT). One risk factor for developing DVT, as described by Virchow’s triad, is venous stasis, which predisposes to thrombus formation [4,5]. Here, we describe an adult woman with an approximately 14-year history of persistent left lower-extremity swelling and pain and an incidental finding of a duplicated IVC. Written consent was obtained from the patient for publication of this case report.

## Case presentation

A 46-year-old woman was referred to our vascular surgery clinic with a history of chronic swelling and pain in the left lower extremity. The symptoms had been present for approximately 14 years. The swelling and pain were localized circumferentially from the inguinal region to the knee and were aggravated by minor physical activity, prolonged standing, and sitting. She reported mild improvement in her symptoms in the prone or supine position. Her medical history included right renal agenesis and uterus didelphys. The patient denied any recent long-distance travel, hospitalization, surgery, major trauma, malignancy, immobility, or hormonal therapy. Her family history was negative for venous thromboembolism or congenital anomalies. Her social history was negative for smoking, illicit drug use, or alcohol consumption. She was taking Xarelto (rivaroxaban) 20 mg daily for left lower-extremity DVT and tramadol for chronic lower-extremity pain, as prescribed by her primary care physician. A review of systems was negative for fever, chills, chest pain, or shortness of breath.

On physical examination, the left lower extremity had circumferential non-pitting edema from the inguinal region to the knee. Reticular and varicose veins were present. There was no calf tenderness, and the distal pulses were palpable (2+). The contralateral extremity was unremarkable. The patient was not in acute distress. Her lungs were clear to auscultation bilaterally, and her heart had a regular rate and rhythm. The abdomen was non-tender and non-distended, with no palpable masses, and bowel sounds were present.

The patient underwent venous duplex USG, which showed iliofemoral DVT. Initially, we proceeded with a conservative approach consisting of elastic graduated compression stockings (EGCS), continued systemic anticoagulation, and follow-up after three months with a non-invasive venous duplex scan. At the follow-up appointment, there was no improvement in her symptoms. The venous duplex scan showed chronic left iliofemoral DVT. Therefore, an elective left lower-extremity venogram was scheduled.

**Figure 1:**
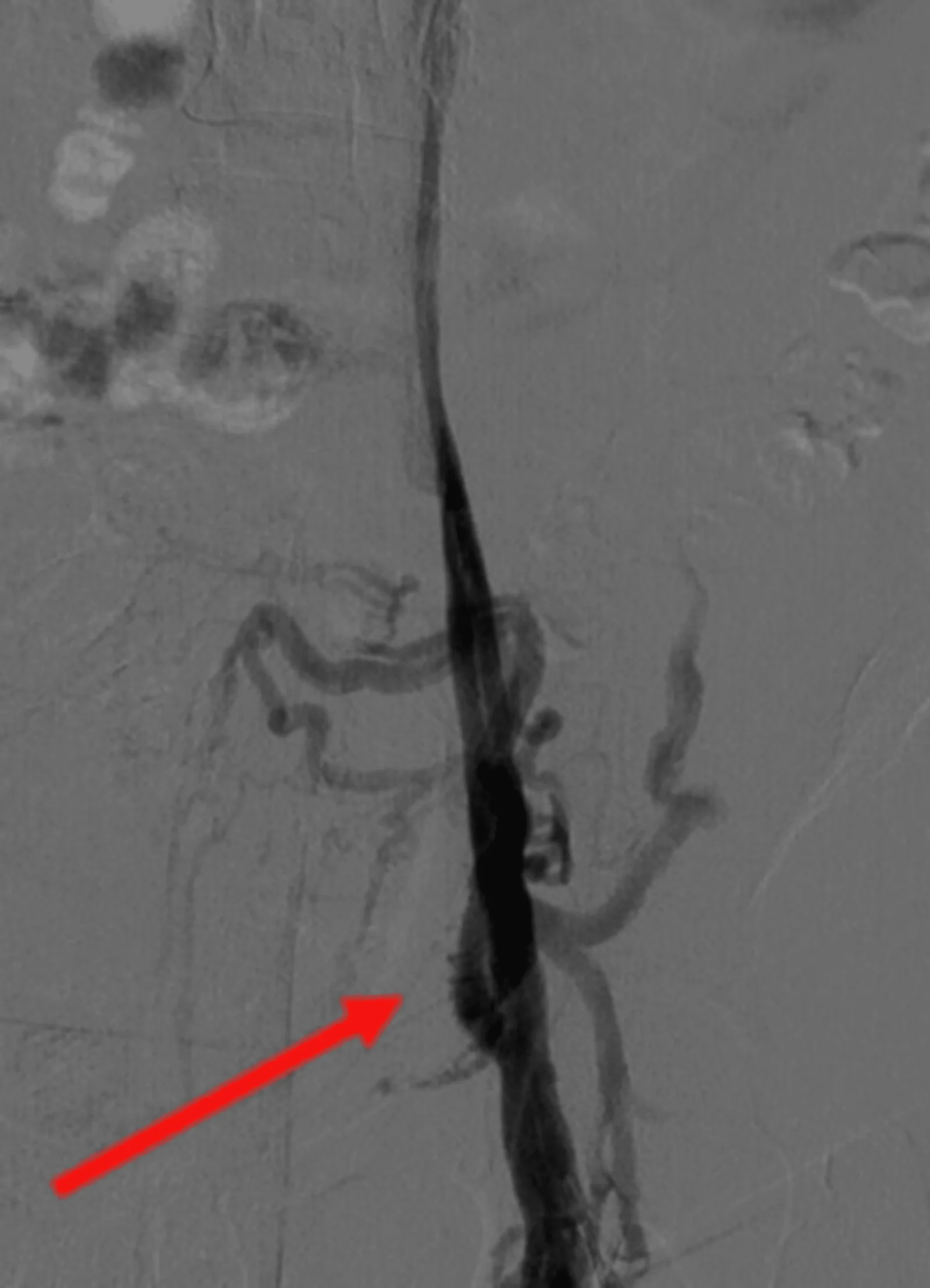
Inferior vena cava venogram demonstrating thrombosis of a duplicated left-sided IVC. Venography of the IVC demonstrates thrombosis of a duplicated inferior vena cava ascending along the left side of the abdomen. The red arrow indicates the occluded segment, with absent contrast opacification consistent with thrombotic obstruction. IVC: Inferior vena cava.

**Figure 2:**
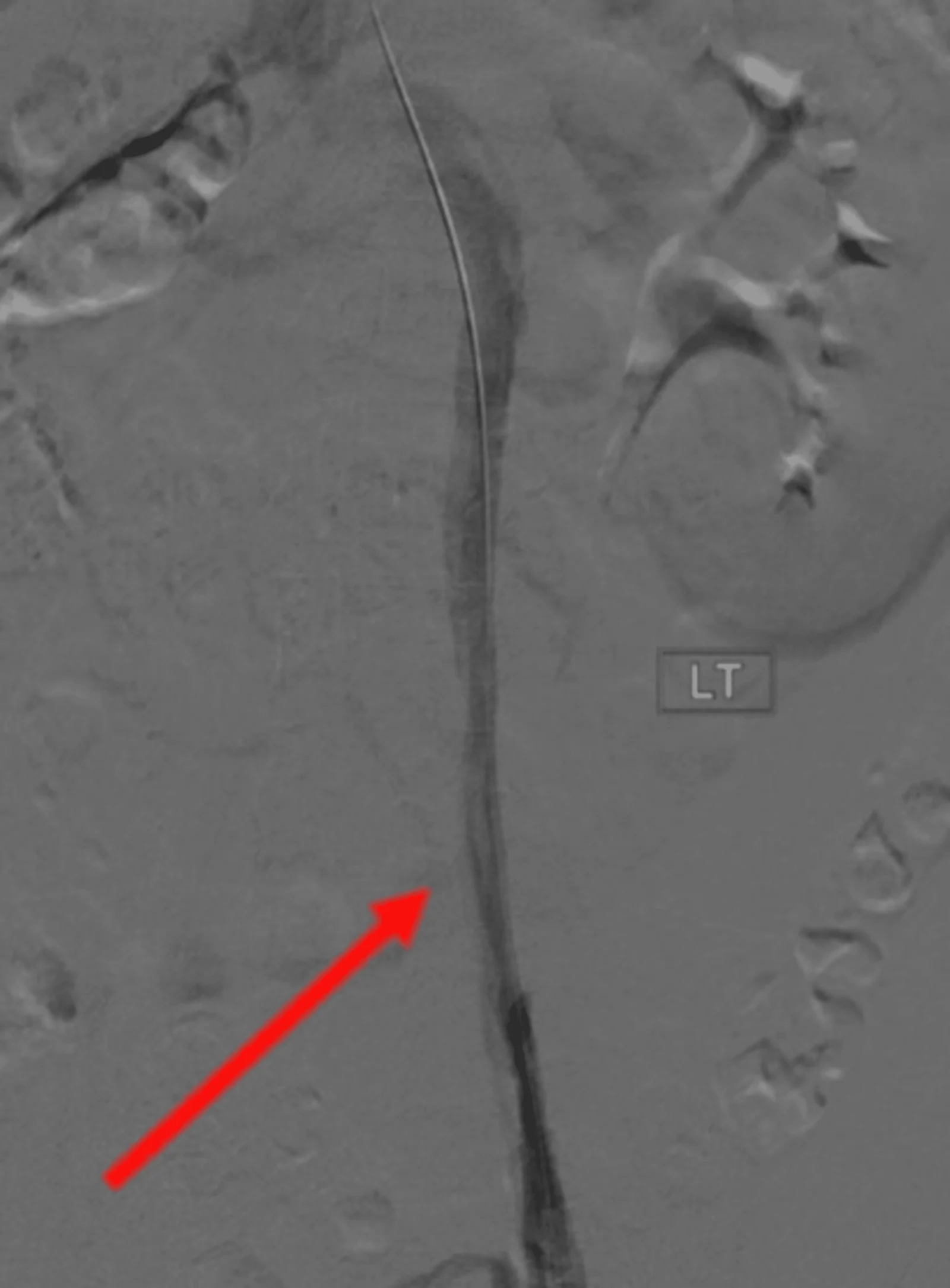
Cannulation and venography of a thrombosed duplicated left-sided IVC. IVC venography following catheter cannulation of a thrombosed duplicated left-sided inferior vena cava. The red arrow highlights the occluded segment, with limited contrast filling confirming persistent venous obstruction. The catheter is visualized traversing the affected vessel. IVC: Inferior vena cava.

**Figure 3:**
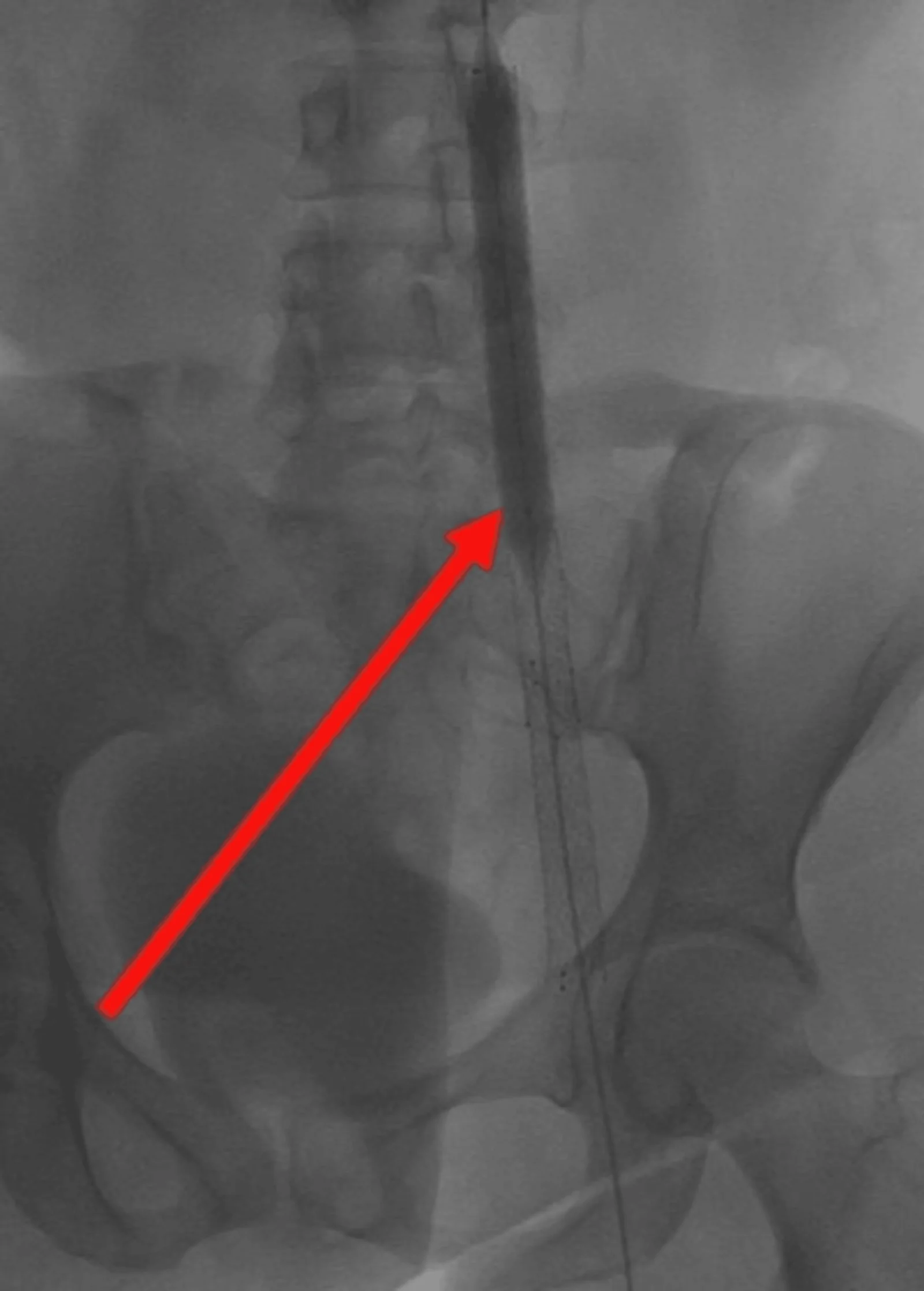
IVC venogram demonstrating intraluminal dilation after angioplasty and stent deployment. Venography demonstrates intraluminal dilation of the IVC, left common iliac vein, left external iliac vein, left femoral vein, and left popliteal vein following endovascular angioplasty and stent deployment. Improved luminal caliber and contrast opacification are noted, consistent with restored venous patency. The red arrow indicates the treated segment. IVC: Inferior vena cava.

**Figure 4:**
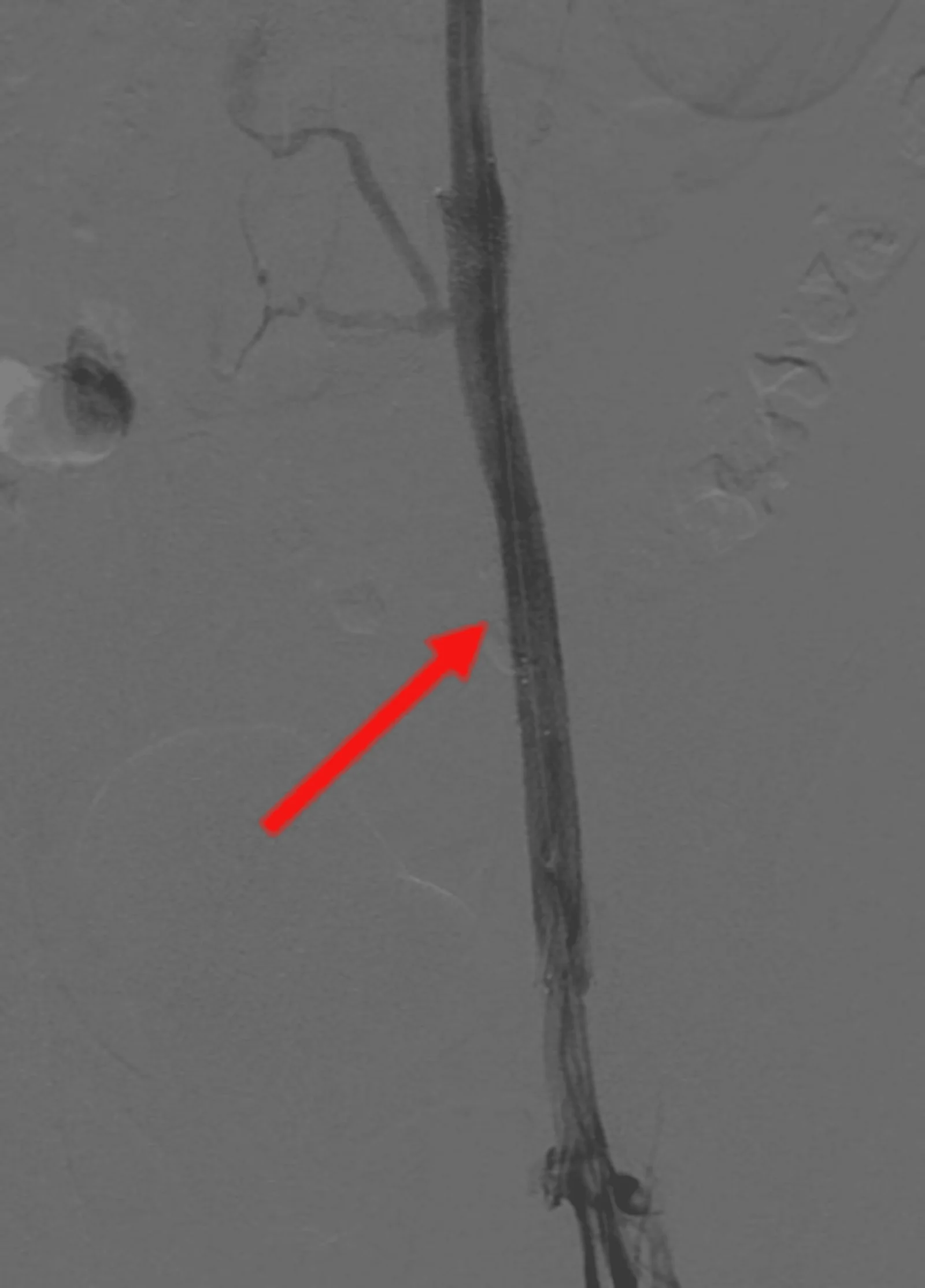
Completion venogram after endovascular stent deployment. The completion venogram obtained following endovascular stent deployment shows restored luminal patency and uniform contrast opacification, consistent with successful recanalization and restoration of venous outflow. The red arrow indicates the stented segment.

**Figure 5:**
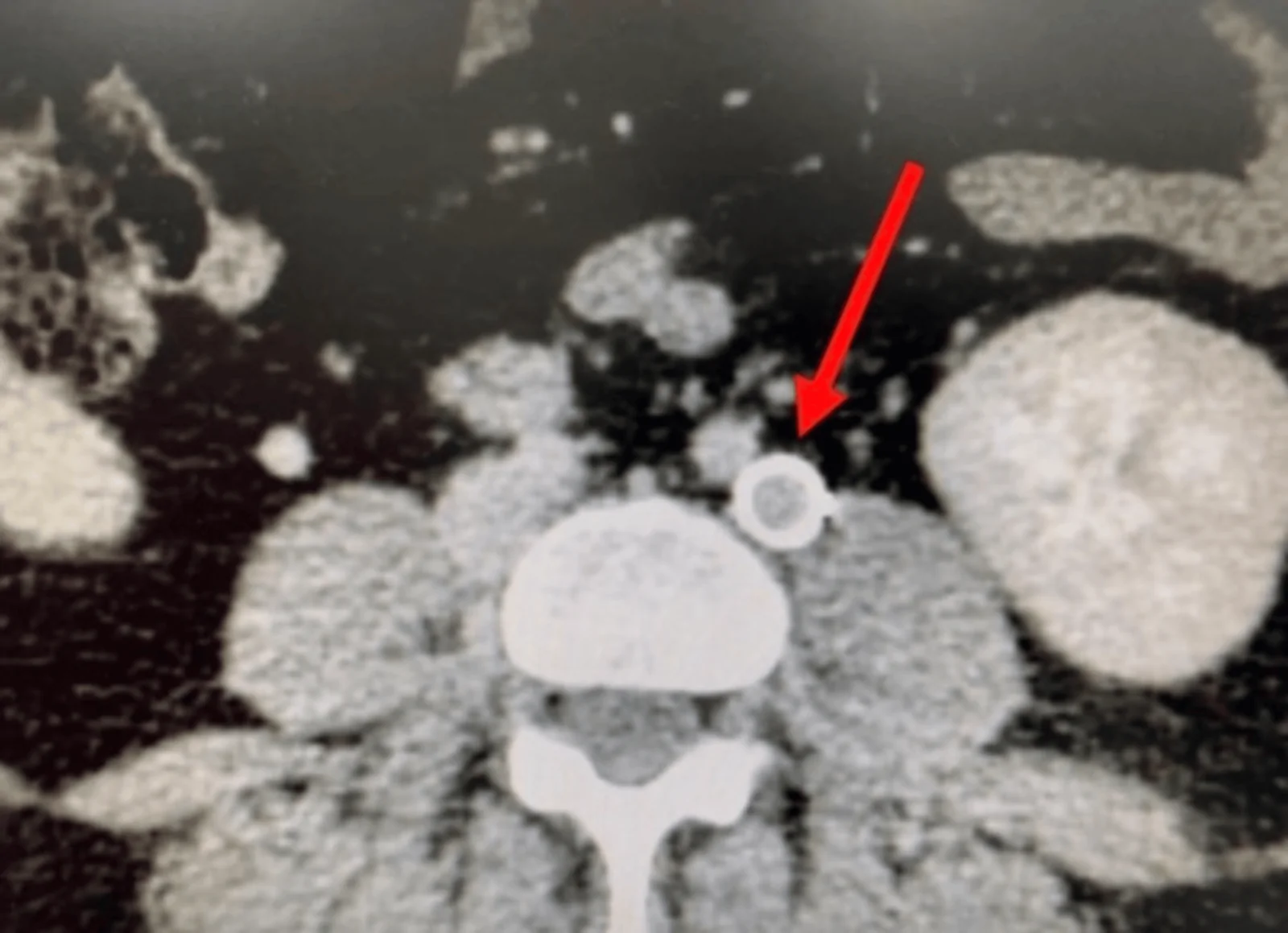
Computed tomography angiography demonstrating stent placement in a duplicated IVC. Axial computed tomography angiography of the abdomen demonstrates a patent endovascular stent within the duplicated inferior vena cava. The red arrow indicates the stented left-sided inferior vena cava, with preserved luminal caliber and contrast opacification confirming vessel patency following the intervention. IVC: Inferior vena cava.

The patient was taken to the operating room, where she underwent the scheduled procedure. Vascular access was obtained through the left popliteal vein under ultrasound guidance. The initial venogram showed a patent orthotopic IVC and a thrombosed duplicated inferior vena cava (DIVC) (Figure 1). The DIVC was selectively cannulated, and additional digital subtraction angiography (DSA) was performed to better visualize the area to be treated (Figure 2). An intravascular ultrasound catheter was used to obtain images from the popliteal vein to the IVC. A 6 × 250 mm balloon was then used to perform angioplasty of the IVC, left common iliac vein (CIV), left external iliac vein (EIV), left femoral vein, and left popliteal vein (Figure 3). Stenting of the IVC, left CIV, and left EIV was performed using 12 × 40 mm, 12 × 120 mm, and 10 × 60 mm stents, respectively. A completion venogram showed patency and adequate placement of the stents (Figure 4). The postoperative course was unremarkable, and no complications occurred. Computed tomography angiography performed at the 2-week follow-up demonstrated the stent within the DIVC (Figure 5). The patient remained asymptomatic at follow-up visits 3 and 6 months after the endovascular intervention.

## Discussion

Among all anomalies of the vena cava system, duplication of the IVC is the most common; however, its reported prevalence is only 0.2-3% in the general population [6,7]. Embryological development of the IVC begins as early as the sixth week of fetal development [1]. It develops from three pairs of venous structures: the subcardinal, supracardinal, and posterior cardinal veins. The suprarenal segment of the IVC is derived primarily from the right subcardinal vein, whereas the infrarenal segment arises primarily from the right supracardinal vein [6,8]. Selective anastomosis and regression of these venous structures eventually give rise to the orthotopic IVC. Abnormalities during embryonic development, such as persistence of both the right and left supracardinal veins, as in our patient, lead to IVC anomalies.

The scientific literature describes various anatomical variants of IVC duplication. Some described variants involve the left IVC draining directly into the left renal vein and then into the right IVC [3,4,9,10]. In contrast, others involve the left IVC and renal vein forming a venous structure that drains into the right IVC [1]. In our patient, the duplicated IVC drained into the left renal vein before emptying into the orthotopic IVC.

In addition to the duplicated IVC, our patient presented with other congenital anatomical anomalies. Uterus didelphys and right renal agenesis were detected on CT imaging. Although other concomitant abnormalities have been described, we found only one other reported case of DIVC with concomitant uterus didelphys [11,12].

German physiologist Rudolf Virchow identified stasis, endothelial injury, and hypercoagulable states as three critical factors that increase the risk of thrombosis [12]. After a thorough literature review, we found that many patients with DIVC and DVT had coagulopathies that increased the risk of thrombus development. Some patients had coagulopathies involving alterations in the coagulation cascade, such as factor V Leiden [4,13,14]. Other patients demonstrated deficiencies in homocysteine metabolism, which increase the risk of coagulation and thrombosis [13]. Upon evaluation, our patient lacked any of these risk factors that could lead to a hypercoagulable state and explain thrombosis of the DIVC. Instead, in our case, we propose that disruption of venous flow in the duplicated IVC led to thrombosis of the affected vein. The majority of cases of DIVC-associated DVT were detected during acute thrombosis of the abnormal vein [3,4,13,14].

Information on the diagnosis and management of DIVC thrombosis is limited because the anomaly itself is usually asymptomatic. We suggest that contrast-enhanced CT should be the imaging study of choice for detecting IVC duplication because it is less invasive and more accessible than venography. Many studies have diagnosed DIVC using CT with IV contrast before performing venography to confirm and treat the anomaly [3,14].

In terms of management, there are currently no guidelines for adequately managing patients with thrombosis of a DIVC. Our initial conservative management was insufficient to successfully treat this patient, and stenting of the duplicated IVC proved to be curative. Low SW et al. initially managed a duplicated IVC DVT with anticoagulation alone, which eventually failed. They opted for stenting of the DIVC, which led to complete resolution of symptoms. Other studies have also shown that stenting of the DIVC was sufficient to manage DVT of the anomaly and prevent future thrombus development [3,4,13,14].

## Conclusions

The duplicated IVC is the most common anomaly of the caval system, yet its complication by DVT is rare. Most patients who present with acute thrombosis of a duplicated vena cava have associated thrombophilia or other coagulopathic disorders; however, our patient lacked other prothrombotic risk factors. The gold standard for diagnosing this condition is venography, but a CT scan with IV contrast is a practical and less invasive diagnostic tool. As in other reported cases, our patient failed initial treatment with oral anticoagulation alone, and her symptoms resolved only after stenting of the DIVC. Therefore, we suggest that a CT scan with IV contrast should be performed in patients with an unprovoked left iliofemoral DVT to rule out caval abnormalities, as in our case.
